# Faecal Microbiota Composition in Adults Is Associated with the *FUT2* Gene Determining the Secretor Status

**DOI:** 10.1371/journal.pone.0094863

**Published:** 2014-04-14

**Authors:** Pirjo Wacklin, Jarno Tuimala, Janne Nikkilä, Sebastian Tims, Harri Mäkivuokko, Noora Alakulppi, Pia Laine, Mirjana Rajilic-Stojanovic, Lars Paulin, Willem M. de Vos, Jaana Mättö

**Affiliations:** 1 Finnish Red Cross Blood Service, Helsinki, Finland; 2 Institute of Biotechnology, University of Helsinki, Helsinki, Finland; 3 Department of Veterinary Biosciences and Department of Bacteriology and Immunology, University of Helsinki, Helsinki, Finland; 4 Laboratory of Microbiology, Wageningen University, Wageningen, The Netherlands; University of Glasgow, United Kingdom

## Abstract

The human intestine is colonised with highly diverse and individually defined microbiota, which likely has an impact on the host well-being. Drivers of the individual variation in the microbiota compositions are multifactorial and include environmental, host and dietary factors. We studied the impact of the host secretor status, encoded by *fucosyltransferase 2* (*FUT2*) -gene, on the intestinal microbiota composition. Secretor status determines the expression of the ABH and Lewis histo-blood group antigens in the intestinal mucosa. The study population was comprised of 14 non-secretor (*FUT2* rs601338 genotype AA) and 57 secretor (genotypes GG and AG) adult individuals of western European descent. Intestinal microbiota was analyzed by PCR-DGGE and for a subset of 12 non-secretor subjects and 12 secretor subjects additionally by the 16S rRNA gene pyrosequencing and the HITChip phylogenetic microarray analysis. All three methods showed distinct clustering of the intestinal microbiota and significant differences in abundances of several taxa representing dominant microbiota between the non-secretors and the secretors as well as between the *FUT2* genotypes. In addition, the non-secretors had lower species richness than the secretors. The soft clustering of microbiota into enterotypes (ET) 1 and 3 showed that the non-secretors had a higher probability of belonging to ET1 and the secretors to ET3. Our study shows that secretor status and *FUT2* polymorphism are associated with the composition of human intestinal microbiota, and appears thus to be one of the key drivers affecting the individual variation of human intestinal microbiota.

## Introduction

Microbiota colonising the human intestine maintains homeostasis and has a marked effect on the human health. This vital role of intestinal microbiota is carried out by a numerous and diverse collection of microbial species that varies from person to person. The drivers of high inter-individual variation are poorly known, but contribution by diet, environment, and host genetics has been proposed. The composition of this complex intestinal microbiome is preserved in an individual for periods longer than a decade [1], indicating that the host has mechanisms for regulating composition and activity of its microbiota. Further, monozygotic twins have been found to carry a more similar intestinal microbiota than dizygotic twins, unrelated persons or family members [2], [3], suggesting a role for host genetics in maintaining the homeostasis. However, other studies have reported that even though monozygotic twins had more similar intestinal microbiota composition than unrelated subjects, their microbiota composition does not differ from that of dizygotic twins [4], [5]. While technical explanations relating to the depth of the analysis cannot be excluded, this has been interpreted as an indication of an effect of shared environment. A better delineating of the impact of host genetics on the intestinal microbiota is of great importance for understanding the relation between microbiota and health as well as for paving the way for personalised treatments for intestinal and other disorders [6].

One candidate host gene affecting the human microbiota is *fucosyltransferase 2* (*FUT2*) gene, which encodes fucosyltransferase 2 enzyme. *FUT2* is responsible for the synthesis of the type 1 H antigens, which act as precursors for the ABO and Lewis b histo-blood group antigens expressed on intestinal mucosa and other secretions. Approximately 20% of individuals of European descent represent the non-secretor phenotype, which is caused by the *FUT2* single nucleotide polymorphism (SNP) rs601338 (W143X, G428A). Non-secretors are homozygous for non-functional *FUT2* (genotype AA), and lack ABO histo-blood group antigens in secretions. Secretor individuals have either one (genotype AG) or two (genotype GG) functional *FUT2* alleles, allowing the synthesis of the ABO antigens in secretions. Recently, secretor status was associated with the composition of human intestinal bifidobacteria [7] and with microbiota composition of humanized gnotobiotic mouse [8].

The non-secretor phenotype or the *FUT2* genotype AA has been associated with several diseases, for example with an increased risk for Crohn's disease [9], type 1 diabetes [10], experimental vaginal candidiasis [11], and urinary tract infections [12]. Secretor status and carbohydrate availability in the intestine have shown to influence the expansion of antibiotic treatment related enteric pathogens, such as *Salmonella* and *C difficile* [13]. Further, Rausch et al. [14] showed that microbiota composition differences related to the *FUT2* polymorphism contributed to the higher susceptibility of the non-secretors to Crohn's disease. Thus, knowledge of the influence of the *FUT2* gene on microbiota composition is highly relevant for the understanding of the aetiology of diseases involving host-microbe interactions.

To determine the impact of the *FUT2* polymorphism on the intestinal microbiota, we analysed the faecal microbiota of non-secretors and secretors by three methods; denaturing gradient gel electrophoresis (DGGE), 16S rRNA gene pyrosequencing and HITChip phylogenetic microarray analysis. The evidence from all of these fundamentally different methods indicated that microbiota compositions differed between the non-secretors and the secretors as well as between the *FUT2* genotypes. Further, the results indicated that the soft clustering of microbiota composition into our representation of enterotypes 1 and 3 was associated with the secretor status/*FUT2* genotype. The overall differences support the conclusion that *FUT2* is one of the host genetic factors explaining individual variation in the microbiota composition.

## Results

### Microbiota composition by DGGE analysis

**Figure 1:**
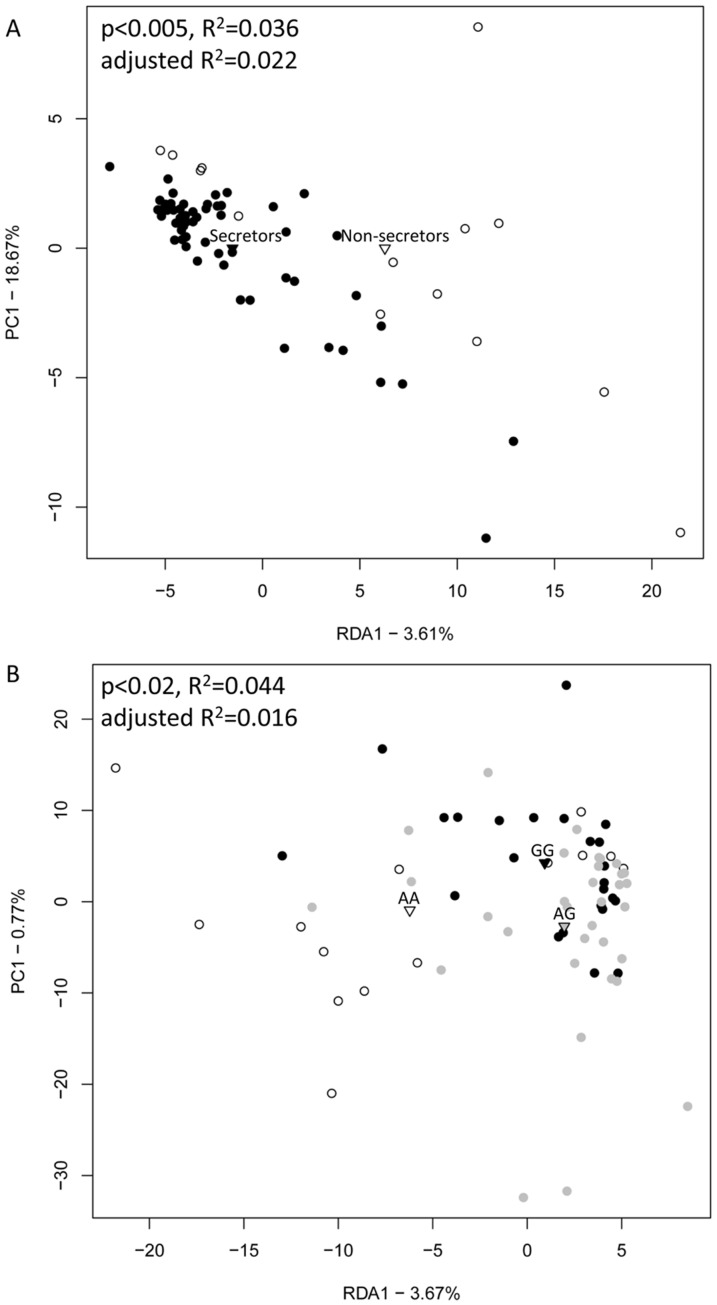
RDA plots of dominant microbiota composition in the secretors (black) and the non-secretors (white) (A) and the *FUT2* genotypes, AA (white), AG (grey) and GG (black) (B). The plots are based on the PCR-DGGE analysis with universal primers. The centroids of each group are indicated by triangles. P-values show statistical significance in ANOVA test. The RDA plots of DGGE analysis with the specific bacterial groups are shown in Figures S1 and S2.

The faecal samples of the 71 individuals were analysed by a PCR-DGGE with several group-specific and universal bacterial primers to profile the intestinal microbiota in the samples. The RDA of the PCR-DGGE profiles revealed that the dominant microbiota composition (ANOVA, F = 2.58, p<0.005) and the composition of bifidobacteria (F = 4.07, p<0.005) and lactobacilli (F = 2.35, p<0.01) differed between the 14 non-secretors and the 57 secretors as well as between the *FUT2* genotypes AA (14), AG (33), and GG (24) (Figure 1, S1, S2). Furthermore, a trend-like pattern was observed for the difference in the *Clostridium* clusters IV (F = 1.51, p<0.07) and XIVa (F = 1.88, p<0.08) between the non-secretors and the secretors (Figure S1). The unconstrained MDS clustering did not show clear clustering in regard to secretor status or FUT2 genotype (Figure S3). Diversity estimates did not differ between the non-secretor and the secretor phenotypes or the *FUT2* genotypes except for bifidobacteria. Bifidobacterial results were reported previously [7]. The diversity estimates and microbiota compositions evaluated by PCA and RDA (data not shown) for a subset of 12 secretor samples included in the in-depth microbiota profiling (see below), were comparable to all secretor samples of the data set (n = 57).

### Microbiota composition analysed by pyrosequencing

The microbiota association with the secretor status and *FUT2* genotype was studied in more detail for the subset of 24 samples (12 non-secretors, AA genotype and 12 secretors of which 5 carried genotype GG and 7 carried genotype AG) by pyrosequencing. Out of the 245 804 generated sequences, 127 352 sequences (52% of reads) passed the quality criteria. The average total read number, Good's coverage and the rarefaction curves were similar between the non-secretors, secretors, and *FUT2* genotypes (Table S1, Figure S4).

**Figure 2:**
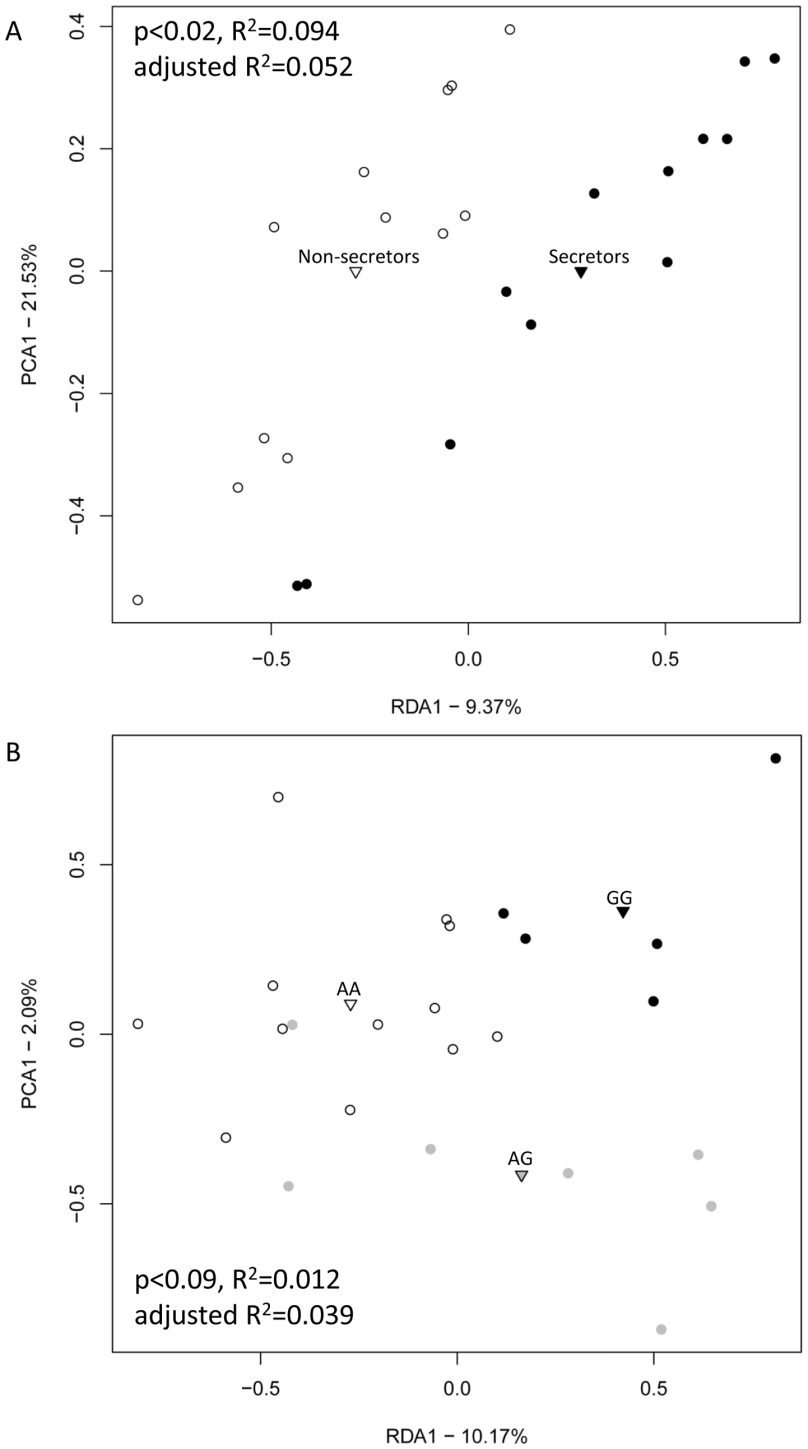
RDA plots of microbiota composition in the non-secretors (white) and the secretors (black) (A) and among *FUT2* genotypes, AA (white), AG (grey) and GG (black) (B). Plots are calculated from the relative abundances of taxa obtained by the 16S rRNA gene pyrosequencing. The centroids of each group are indicated by triangles. P-values show statistical significance in ANOVA test.

RDA based on the observed genera (ANOVA, F = 2.37, p = 0.02) and OTUs (F = 1.34, p = 0.02) showed statistically significant differences in the microbial composition between the non-secretors and the secretors (Figure 2 and S5). Similar microbiota differences in relation to secretor status was observed in RDA with subsampled dataset (F = 1.93, p = 0.02) (Figure S6), which standardize the sequencing coverage between the samples, indicating that the differences were not due to variation in sequence coverage. In addition, AMOVA indicated a statistical significance for the difference between the non-secretors and the secretors (p = 0.02). Between the *FUT2* genotypes, a trend-like difference (p = 0.08) was found by AMOVA and RDA on the genera (F = 1.47, p = 0.09) (Figure 2). RDA based on the OTUs or the subsampled dataset showed no significant differences between the *FUT2* genotypes (Figures S5 and S6). Neither did MDS show clear clustering between the secretors and the non-secretors or between the *FUT2* genotypes (Figure S3).

**Figure 3:**
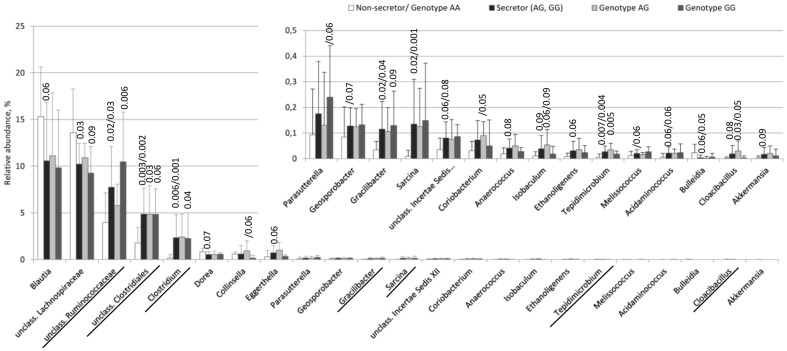
Mean relative abundances of the bacterial genera showing significant (ANOVA/indicator species analysis, p<0.05) or trend-like differences (p<0.09) between the non-secretors (n = 12) and the secretors (n = 12) or between the *FUT2* genotypes AA (n = 12), AG (n = 7), GG (n = 5). The statistical significance is indicated for a comparison of the secretors/*FUT2* genotypes AG and GG to the non-secretors/*FUT2* genotype AA. The taxa significantly differing in both indicator species analysis and ANOVA test are underlined.

To reveal the bacterial taxa contributing to the altered microbiota composition, the relative abundances of the taxa were compared in relation to the secretor status and the *FUT2* genotypes. As expected, the microbiota composition varied greatly in both the non-secretors and secretors (Figure. S7). At the phylum level, the non-secretors, secretors and *FUT2* genotypes shared similar microbiota compositions, dominated by Firmicutes (on average 93% of the total microbiota) (Figure S7). Only 4%, 2% and 0.4% of the sequences were classified into Bacteroidetes, Actinobacteria and Proteobacteria, respectively. Yet relative abundances of the six bacterial genera, such as *Clostridium*, and genera belonging to families *Ruminococcaceae*, and unclassified Clostridiales varied notably between the non-secretor and the secretors by both the ANOVA test and the indicator species analysis (Figure 3). Several other genera, including the mucus-degrader *Akkermansia* [15], showed a trend towards a lower relative abundance in the non-secretors (ANOVA, F = 3.05, p = 0.09) (Figure 3). The abundance of *Akkermansia* (13 sequences) and several other taxa were close to the detection level with our sequencing depth, and thus a single sample may contribute greatly to the effect. The associations of genera with the *FUT2* genotypes/secretor status were weak, and their significances disappeared when the p-values were corrected for the number of performed statistical tests.

The average richness of genera was lower in the non-secretors (on average 71 genera/sample) in comparison to the secretors (on average 84 genera/sample) by c2m randomization test (p = 0.04), which was verified with subsampled data (on average 58 and 69 genera/sample in the non-secretors and the secretors, p = 0.02). The average richness of OTUs did not differ between the non-secretors and the secretors in c2m test (1298 vs. 1498, p = 0.104). At the genera level, mean inverse Simpson and Shannon diversity indeces were lower in the non-secretors than in the secretors (inverse Simpson: 9.0 vs. 11.8, ANOVA, F = 4.24, p = 0.05; Shannon: 2.6 vs. 2.9, ANOVA, F = 4.54, p = 0.04), but these border line differences were not verified using the subsampled or OTU datasets. Diversity, average richness or rarefaction curves (Figure S4) did not differ between the FUT2 genotypes, nor were such differences detected in the rarefaction analysis based on genera or OTUs between the non-secretors and the secretors (Figure S4). In total, 215 bacterial genera were detected in the whole dataset. No statistically significant co-occurrences were identified in the analysis of pyrosequencing data.

### Phylogenetic microarray analysis

**Figure 4:**
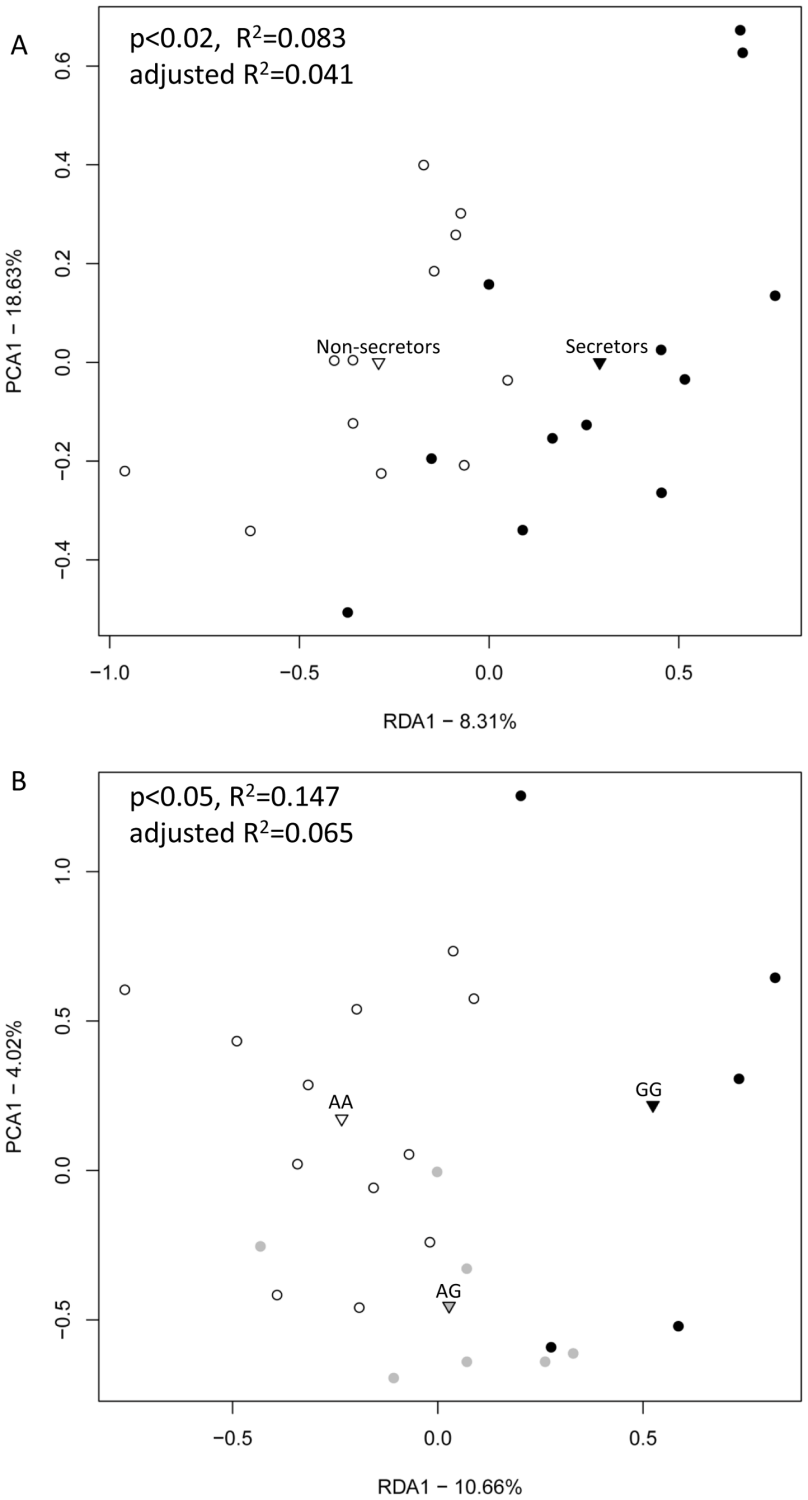
The RDA plots of microbiota composition in the non-secretors (white) and the secretors (black) (A) and among the individuals with *FUT2* genotypes, AA (white), AG (grey) and GG (black) (B). The RDA plots were based on genus-like/level 2 intensities of HITChip analysis. The centroids of each group are indicated by triangles. P-values show statistical significance in ANOVA test.

The 24-sample subset was analysed additionally by the phylogenetic microarray, the HITChip. The RDA on relative abundances of HITChip level 2/genus-like taxa, showed significant differences in microbiota compositions between the non-secretors and the secretor samples (F = 1.99, p<0.03) as well as between the *FUT2* genotypes (F = 1.81, p<0.05) (Figure 4). Similar results were obtained with level 3 taxa (species-like level) (secretor status F = 1.51, p<0.06; genotypes F = 1.40, p<0.05) (Figure S8). Moreover, unconstrained clustering by MDS based on level 2 taxa showed clustering of the samples in relation to the *FUT2* genotypes/secretor status (Figure S3). The soft classification of the samples into enterotypes, and the effect of the secretor status/genotype on the classification were assessed with HITChip level 2 data. No samples belonging to the ET2 were present in our dataset but 6 samples had microbiota composition resembling the ET1 and 18 samples had composition resembling the ET3. The dependency between secretor status/genotype and the soft classification of samples into ET1 (Kruskal-Wallis; secretor status χ^2^(1) = 7.84, p = 0.005; genotype χ^2^(2) = 7.86, p = 0.02) and ET3 (secretor status χ^2^(1) = 7.52 p = 0.006; genotype χ^2^(2) = 7.56, p = 0.03) was statistically significant. The abundances of enterotype indicator species in the secretors, non-secretors and *FUT2* genotypes are shown in Table S2.

Based on the HITChip hybridisation signals, a significant reductions in the mean inverse Simpson and Shannon diversity indices were evident for the non-secretors, when compared with the secretor phenotype (ANOVA, Inverse Simpson index F = 13.4, p = 0.001; Shannon index F = 14.47, p = 0.001) (Figure S9).

By HITChip, intestinal microbiota of both the non-secretors and the secretors was dominated by Firmicutes (84%), followed by Bacteroidetes (11%) and Actinobacteria (2.4%). The relative abundances of several levels 1, 2, and 3 taxa, mainly belonging to the dominant phyla Firmicutes, were significantly influenced by the secretor phenotype and/or the *FUT2* genotypes (Table S3, S4, S5 and S6). The abundances of the taxa differed mainly between the *FUT2* genotype AA (non-secretor genotype) and the homozygous *FUT2* genotype GG, and less between the genotype AA and the heterozygous genotype AG (Tables S3, S4, S5 and S6). These included the taxa of bifidobacteria, *B. angulatum*, *B*. *catenulatum* and *Bifidobacterium* spp., which were significantly more abundant in the *FUT2* genotype GG than in the genotype AA, a non-secretor genotype (Table S6). The histogram indicated a clear accumulation of significant p-values, increasing the evidence for the association of the secretor status or the *FUT2* genotype with microbiota (Figure S10). Similarly to the sequencing data, the associations of the taxa with the *FUT2* genotype or the secretor phenotype were rather weak in the HITChip analysis and the correction for the number of performed statistical tests resulted in the disappearance of the significant p-values.

The analysis of co-occurrences of taxa based on Spearman correlation in the secretors and the non-secretors revealed that seven bacterial taxa pairs co-occurred in the secretors, even when applying a stringent threshold to the statistical significance (q-values <0.05) (Figure S11). In the non-secretors, a single positive co-occurrence was observed (Figure S11). The correlations between the microbial groups calculated as Pearson correlation coefficients (on log transformed data) showed the same trends as those with Spearman (data not shown), the only difference being that fewer anti-correlations where detected using Pearson. Especially at the higher level phylogeny, similar amounts of connections were formed in both the secretors and the non-secretors.

## Discussion

Our findings based on the microbiota profiling with three methods (PCR-DGGE, pyrosequencing and the HITChip) showed that the composition of the intestinal microbiota was associated with the secretor status and with the determinant of the secretor status, the *FUT2* gene polymorphism. The secretor status/*FUT2* appeared to be one of the host genetic features contributing to the human microbiota and inter-individual microbiota variation. This demonstrates that a single host gene can have a significant effect on the intestinal microbiota composition in healthy adults.

The human intestinal microbiota has recently been tentatively clustered into three main types, called enterotypes, suggested to be characterised by a few key genera [16]. The drivers of these clusters, which are still under debate [17], [18], are not fully known, but diet, environment and host genetics have been proposed. Indeed, long-term diet has been reported to explain part of the enterotypes [19] and immunity-related host gene GTPase family M gene (IRGM) (SNP rs1747270) has been associated to enterotype clustering (Quince et al, 2013). In the study of microbiota in obese and lean monozygotic twins [3], showed that twin pairs, even when discordant in terms of body mass index, shared highly similar microbiota, and concluded that human microbiota was strongly linked to the host genotype. Moreover, about half of the genera representing the core microbiota of twins (so called structural core) overlapped with the reported key genera of enterotypes, indicating that genotypic factors may be drivers of enterotype clustering [3]. We treated the enterotypes here as examples of certain observable states in microbiota, since their very existence and interpretation as true universal discrete representations of microbiota are still under on-going research. Nevertheless, using HITChip data, we evaluated the role of secretor status, which was shown to associate with microbiota composition in this study, as one potential driver of the enterotypes clustering of human microbiome. Samples of this dataset belonged to only two (ET1 and ET3) of the three reported enterotypes. We resorted to a soft classification, in concordance with Huse et al. [17] and Koren et al. [18], who have argued that the enterotypes are more likely to form continuous than distinct clusters. An analysis of the classification results indicated that the non-secretors had a higher probability of belonging to ET1 and the secretors to ET3, observed with a supervised classification method. Although the secretor status did not exclusively explain the enterotype classification, it indicated that secretor status supposedly together with other factors, such as long-term diet [19] and IRGM [20] may affect the intestinal microbiota composition among individuals. Recently, several methodological aspects (eg. clustering method and distance metric) were shown to affect enterotype clustering strength [18]. This may also have influence by clustering of Arumugam et al. [16], which our classification of the samples was based on. Summarizing, this analysis provides further evidence that microbiota and genotype, in this case secretor status, are strongly linked.

Several autoimmune diseases such as the inflammatory bowel disease (IBD) [9], [21], and type I diabetes [10] have been associated with the *FUT2* polymorphism. By studying microbiota of IBD patients with known *FUT2* genotypes Rausch et al. showed that the *FUT2* genotype could contribute to the susceptibility for IBD through altered microbiota composition [14]. The present study comparing the fecal microbiota in 14 non-secretors and 57 secretors showed that even healthy non-secretors and secretors have differences in their intestinal microbiota. Similarly, differences in composition of mucosal microbiota between healthy non-secretors and secretors were suggested in study by Rausch et al. on the basis of only three healthy non-secretors [14]. Actually, we observed that the bacteria belonging to *Blautia* et rel., *Dorea formicigenerans* et rel., *Ruminococcus gnavus* et rel., and *Clostridium sphenoides* et rel., were significantly more abundant in the non-secretors than in the secretors. Interestingly, all these taxa have been associated with IBS and/or IBD in several studies [22]–[24] and may thus indicate that non-secretors have propensity for intestinal aberrations. In addition to IBD, higher susceptibility to e.g. coeliac disease [25] and diabetes type 1 [26] has been associated with the dysbiosis of microbiota and in separate studies also with non-secretor status [10], [27]. Based on these examples it is tempting to speculate that the *FUT2* genotype may be a relevant factor that induces alterations in the microbiota composition and plays a role in aetiology of the several diseases involving host-microbiota interactions.

Intestinal bacterial species are commonly equipped with varying glycan degrading enzymes and abilities to access mucus glycans, allowing them to target and benefit from the complex glycan structures, such as the ABO histo-blood group antigens, present in the intestine [28]. The interaction between the bacteria and the fucosylated glycan antigens of the host has been elegantly demonstrated for different *Bacteroides* species [29]. The two Bacteroides taxa co-occurring in the secretors in this study, *B. plebeius* et rel. and *B. fragilis* et rel., have a very high number of genes coding for α-L-fucosidase (>10 genes/genome) and (8–10 genes/genome) β-galactosidase enzymes, which potentially enables them to utilize of ABO glycan structures and take advantage of this constant source of growth substrates on intestinal mucosa. Enzymes for the degradation of the ABO glycan structures have also been identified in *Bifidobacterium* species and the mucus degrading genera *Ruminococcus*, *Clostridium* and *Akkermansia* [30]–[35], which were among the significantly abundant bacteria in the secretors in this study. In secretors, these genera co-occurred with bacteria (*Sporobacter, Lachnobacillus*, and *Oxalobacter*), which do not have enzymes for the ABO antigen degradation in their genomes. For example *Akkermansia* have α-L-fucosidases, and β-galactosidases [35] allowing them to remove the terminal and other sugar moieties of the ABO histo-blood group antigens, whereas *Oxalobacter*, unable to grow on sugars [36], could benefit from end products of *Akkermansia*. The co-occurrences indicated that the ABO histo-blood group antigens may benefit through food-web also bacteria incapable of ABO degradation. Besides growth substrates, the ABO histo-blood group antigens provide adhesion receptors for bacteria as described for several pathogens such as *Helicobacter pylori* [37] and *Staphylococcus aureus* [38] as well as for commensal microbes such as *Lactobacillus gasseri* [39]. In addition to the secretor status/*FUT2*, other genetic factors are likely to influence the intestinal microbiota composition, but the information is still limited. Recent report showed that NOD2 genotypes have an influence also on human microbiome [40]. Several metabolism and immunity related genes, such as leptin, NOD and TLR encoding genes, have shown to co-vary with the mouse microbiome [41].

We showed the difference in the dominant microbiota composition between the secretors and the non-secretors by three profiling techniques. We have previously indicated that in general the HITChip and the pyrosequencing produce comparable results, but the resolution of HITChip has been reported to be higher for intestinal bacterial taxa with low abundance than the resolution of routine pyrosequencing [42], [43]. As an example of the impact of a microbiota profiling technique, we observed different levels of changes in the bifidobacterial population by applying different methods. Our previous results by PCR-DGGE and qPCR methods showed that the composition and diversity of intestinal bifidobacteria was strongly associated with the secretor status [7]. Indeed, the abundances of several bifidobacterial taxa differed between the non-secretor genotype (AA) and the secretor genotype GG (but not AG) according to the HITChip, confirming our previous results on the association of bifidobacteria with the *FUT2* [7]. However, no difference was detected in the diversity of bifidobacteria. The bifidobacterial 16S rRNA genes are closely related, which may cause cross-hybridization and thus, the masking of some differences between non-secretors and secretors. In general, the abundance of Firmicutes, Bacteroidetes, and Actinobacteria differed between Hitchip and pyrosequencing methods applied in this study (84%, 11% and 2,4% by HITChip; 93%, 4% and 2% by pyrosequencing, respectively). The low detection frequency of Bacteroidetes and Actinobacteria by pyrosequencing may be due forward primer F27, which does not allow optimal recovery of Bacteroidetes and Actinobacteria in comparison to Firmicutes. The same DNA extraction method was applied for both methods. A low number of bifidobacteria sequences (2%) by pyrosequencing also indicates that more sequencing effort would have been necessary in order to reveal the bifidobacteria-related differences. The recent mouse study of Kashyap et al. [8] showed that the *FUT2*/secretor status associated changes of intestinal microbiota were diet-dependent. In the present study, information on the dietary habits was not collected and the effect of diet could thus not be investigated.

This study shows that non-secretors have an altered intestinal microbiota community and strengthens the evidence indicating that the *FUT2* polymorphism influence on the intestinal microbiota. The secretor status is one of the drivers of host-associated variation in the microbiota composition and, together with other factors it may contribute to the clustering of microbiota into enterotypes.

## Materials and Methods

### Samples

To study the association of *FUT2* on intestinal microbiota of healthy non-secretor and secretor individuals, faecal and blood samples were collected from 71 adults of European descent, of whom 14 were non-secretors (*FUT2* SNP FUT2 rs601338 genotype AA, 12 Lewis a phenotype, 2 Lewis negative phenotype) and 57 were secretors (33 had genotype AG and 24 genotype GG, all had Lewis b phenotype). Based on the exclusion criteria confirmed by interviewing the volunteers, subjects with clinically diagnosed intestinal diseases or regular intestinal disturbances or antibiotic therapy two months prior to the sampling were excluded. The participants consumed mixed diet without any restrictions, except that the consumption of probiotics was restricted one week before faecal sampling. No other dietary information was collected. The study had the approval of the ethical committee of the Helsinki University Hospital and all the subjects signed a written informed consent. The age, gender and ABO distribution of the participants is shown in [7]. The faecal samples for the microbiota profiling were frozen at −80°C within 5 hours of defecation. EDTA anticoagulated peripheral blood samples for blood group analysis were kept at +4°C and analysed within 24 hours. Buffy coats were extracted from citrate anticoagulated peripheral blood samples by centrifugation and stored at −80°C until DNA extraction.

### Determination of secretor status and genotype

Human DNA was extracted from the buffy coat preparations using the QIAamp DNA Blood Mini Kit (QIAGEN Inc, CA, US). ABO blood groups were determined by serology and by genotyping the *FUT2* SNP rs601338 as described in Wacklin et al. [7].

### Microbiota profiling

DNA for the DGGE analysis was extracted from the faecal samples using the FastDNA SPIN Kit for Soil and the FastPrep Instrument (MP Biomedicals, CA, USA) as described by Wacklin et al. [7]. DNA for pyrosequencing and HITChip analyses was extracted using the method of Apajalahti et al. [44] as described in Mäkivuokko et al. [45].

#### DGGE

All 71 faecal samples were analysed by PCR-DGGE using universal bacterial primers, and bifidobacteria, lactobacilli, *B. fragilis* group, *Clostridium* cluster IV, and cluster XIVa specific primers using Dcode universal mutation detection system (Bio-Rad, CA, USA) as described in [45]. Despite several attempts, PCR was unsuccessful for 7 samples with bifidobacteria primers, two samples with *B. fragilis* specific primers, and one sample with *Clostridium* cluster IV and lactobacilli primers. The DGGE gels were analysed and principle component analyses (PCA) were performed for the secretors and the non-secretor as well as for the individuals with *FUT2* genotypes AA, AG and GG with Bionumerics (Applied Numerics) as described by Wacklin et al. [7]. Diversity estimates, redundancy analysis (RDA) and Multidimensional scaling (MDS) with Bray-Curtis distance and statistical tests (ANOVA) were based on intensity matrixes of the secretors and the non-secretor, and the individuals with different *FUT2* genotype and analysed using R 2.14.2 (R Development Core team, 2012) and its extension package vegan version 2.0–3 [46].

#### The 16S rRNA gene pyrosequencing

For in-depth profiling of microbiota, the bar-coded pyrosequencing method was applied to 24-sample subset, containing the samples of 12 non-secretors (AA genotype carriers) and 12 secretors (5 GG or 7 AG genotype carriers). The subset included all non-secretor samples, except those two Lewis negative individuals. The secretor individuals were matched with the non-secretors according to their ABO blood group, age, and gender. The V1-V3 region of the 16S rRNA gene was PCR amplified in three replicates using a universal bacterial primer pair (F27 5′-AGAGTTTGATCMTGGCTCAG-3′; 518R 5′-ATTACCGCGGCTGCTGG-3′). The PCR products were quantified and pooled in equal amounts and sequenced using a Genome sequencer FLX Titanium (Roche) in the Institute of Biotechnology (University of Helsinki, Finland). The raw sequences (245 806) were trimmed using Mothur v.1.19.0 [47]. The sequences with averaged quality score over 30, length over 300 bases, exact matches to barcode tags and the forward primer, no ambiguous bases, no homopolymers longer than 8 bp, and which were non-chimeric were included in the analysis. The high-quality sequences were binned to the samples according to barcode tags, and into operational taxonomic units (OTUs) applying the furthest neighbor algorithm, threshold of certainty over 60%, and sequence similarity over 97%. The sequences were classified into bacterial taxa using the nearest neighbor method and SILVA release 111 as a reference database [48] and the classify.seqs tool implemented in Mothur [47]. The diversity and richness in the non-secretors and the secretors as well as in the individuals with different *FUT2* genotypes was estimated with several methods: The average species richness was assessed with the R extension package rich version 0.1 [49] using a c2m randomization test with 999 randomisations [50]. Inverse Simpson and Shannon diversity indices were calculated in R extension package vegan. Indicator species analysis [51] using untransformed data was carried out in R using the extension package labdsv version 1.4.-1 [52]. Abundances of OTUs and taxa between the non-secretors and the secretors as well as between the different *FUT2* genotypes were Hellinger transformed [51], [53] and used for RDA with 999 permutations and for MDS applying Bray-Curtis distance using R with package vegan. An analysis of molecular variance (AMOVA) [54], which is a distance-based, non-parametric approach based on permutations to derive the sum of squares and a pseudo F statistic, was conducted in R using the package pegan [55] for the non-secretors, the secretors and the different *FUT2* genotypes. Additionally, RDA, MDS and diversity estimates were analysed using a 2500 sequence subsample from each sample (total 60 000 sequences). Random subsampling was performed in Mothur.

#### HITChip

A phylogenetic microarray analysis by the Human Intestinal Tract (HIT) chip [56], which contains probes for over 1000 known intestinal species and allows also the detection of the taxa present in low numbers [22], was applied to the 24-sample subset. The hybridisation of the samples was performed two times with a reproducibility of >98% (assessed by Pearson's correlation coefficient). The HITChip analysis was performed as earlier described in [56]. In short, the data was quality controlled, within-array spatial normalization was performed, outliers were removed, and quantile normalisation was applied to the results of two hybridisations of the same sample [56]. Min-max normalization as described in [56] was used for the calculation of the inversed Simpson and Shannon diversity indices on level 3 data in R package vegan. For all the other analyses between-array normalization was performed with quantile normalization [57]. The differences in each bacterial group between the non-secretors and the secretors as well as between the different FUT2 genotypes were analysed with linear models and ANOVA tests, transforming the array intensities into logarithmic scale. RDA and MDS analyses were based on Hellinger transformed data and analysed similarly to pyrosequencing data. The enterotype classification was performed based on the HITChip data of the samples, combined with the HITChip data for all the MetaHIT samples (n = 124) classified originally by Arumugam et al. [16], which was used as a training set. Three distinct groups, so called Enterotypes, were found [16]. The abundances of genera in the non-secretor and secretor samples were compared against a set of predictive models (*ntree*  = 1000), which were built while using the training set, in a random forest approach [58]. Probability scores of the classification were estimated from random forest models from the training set. The association of enterotypes with secretor status and genotypes was measured with Kruskal-Wallis test using the probability of belonging to a certain enterotype and the host secretor status/*FUT2* genotype. Co-occurrence networks were built using Spearman correlations based on relative abundances and Pearson correlations based on log transformed relative abundances of the level 2 taxa, using thresholds of ρ>0.7, relative abundance of the taxa >0.1%, and presence in >50% of the non-secretors or secretors. The networks were visualized using the Gephi network visualization and exploration platform [59]. The q-value threshold for significant co-occurrence was set at 0.05. The original HITChip data that was used for our analysis is in table S7.

## Supporting Information

Figure S1 **RDA plots of bifidobacteria, lactobacilli,** ***Clostridium*** **cluster IV and XIVa and** ***Bacteroides fragilis*** **populations in the non-secretors (white) and the secretors (black).** The RDA analyses were based on PCR-DGGE profiles of the samples. The centroids of each group are indicated by triangles. P-values show statistical significance in ANOVA test.
(PDF)

Figure S2 **RDA plots of bifidobacteria, lactobacilli,** ***Clostridium*** **cluster IV and XIVa and** ***Bacteroides fragilis*** **populations in the individuals with** ***FUT2*** **genotypes AA (white), AG (grey) and GG (black).** The RDA analysis based on the PCR-DGGE profiles of the samples. The centroids of each group are indicated by triangles. P-values show statistical significance in ANOVA test.
(PDF)

Figure S3 **MDS plots of intestinal microbiota compositions in the individuals with FUT2 non-secretor genotype AA (white), secretor genotype AG (grey) and GG (black) based on band intensities of the DGGE analysis (A), on abundances of genera by pyrosequencing (B), on abundances of level 2 taxa by HITChip (C), on abundances of OTUs by pyrosequencing (D) and on abundances of genera in subsampled pyrosequencing data.**
(PDF)

Figure S4 **Rarefaction curves for the non-secretor and the secretor samples based on detected OTUs using 0.97 similarity threshold (A) and on the genera (B).** Blue line =  non-secretors/FUT2 genotype AA, red line =  FUT2 genotype AG, green line =  FUT2 genotype GG.
(PDF)

Figure S5 **RDA plots based on the OTUs detected in the non-secretors (white) and the secretors (black) (A) and among the** ***FUT2*** **genotypes, AA (white), AG (grey) and GG (black) (B).** Threshold of 97% similarity was used for clustering of the sequences into OTUs. The centroids of each group are indicated by triangles. P-values show statistical significance in ANOVA test.
(PDF)

Figure S6 **RDA plots of microbiota compositions at genus level in the non-secretors (white) and the secretors (black) (A) and among the** ***FUT2*** **genotypes, AA (white), AG (grey) and GG (black) (B).** Plots are based on random subsample of the pyrosequencing data set (2500 sequences per sample, total 60 000 sequences). The centroids of each group are indicated by triangles. P-values show statistical significance in ANOVA test.
(PDF)

Figure S7 **Relative abundances of bacterial phyla in the secretors/** ***FUT2*** **genotypes AG (n = 7) and GG (n = 5) and in the non-secretors/genotype AA (n = 12).** The abundances were based on the 16S rRNA gene pyrosequencing.
(PDF)

Figure S8 **RDA plots of intestinal microbiota compositions in the non-secretors (white) and the secretors (black) (A) and among individuals with the** ***FUT2*** **genotypes AA (white), AG (grey) and GG (black) (B).** Plots were based on Hellinger transformed level 3/species-like taxa obtained by HITChip analysis. The triangles indicate centroids of the study groups. P-values show statistical significance in ANOVA test.
(PDF)

Figure S9 **Bacterial diversity in the individuals with** ***FUT2*** **non-secretor genotype AA (n = 12), and with secretor genotypes AG (n = 7) and GG (n = 5).** The results were based on the HITChip analysis.
(PDF)

Figure S10 **ANOVA p-value histogram for level 3/species –like level differences between the non-secretor and the secretors (A) and different** ***FUT2*** **genotypes (B) in HITChip analysis.**
(PDF)

Figure S11 **Co-occurrences based on the relative abundances of the level 2/genus-like level bacterial taxa for the secretors (A) and the non-secretors (B).** Co-occurrences with ρ>0.7, relative abundance of the bacterial group >0.1%, and present >50% of the non-secretor or secretor samples are shown. Light green indicate positive and yellow negative co-occurrences. Statistically significant positive and negative co-occurrences (q-value <0.05) are shown in blue and red, respectively.
(PDF)

Table S1 **The number of the 16S rRNA gene sequences obtained from the non-secretor samples, the secretor samples and the samples with different FUT2 genotypes by pyrosequencing.**
(PDF)

Table S2 **Average relative abundances (%) of enterotype indicator species in the non-secretors, the secretors and the individuals with FUT2 genotypes AA, AG or GG belonging to the enterotype 1 (ET1) or 3 (ET3).**
(PDF)

Table S3 **The bacterial level 1 and 2 taxa, whose relative abundances were significantly different (p-value <0.05 in ANOVA) between the non-secretor (NSS) and the secretor (SS) individuals by the HITChip analyses.**
(PDF)

Table S4 **The bacterial level 1 and 2 taxa, whose were relative abundances were significantly different between the samples with different FUT2 genotypes based on HIT Chip analyses.**
(PDF)

Table S5 **The bacterial taxa at the level 3/species-like level, whose relative abundances were significantly different (p-value <0.05 in ANOVA) between the non-secretor (NSS) and the secretor (SS) individuals by the HITChip analysis.**
(XLSX)

Table S6 **The bacterial taxa at the level 3/species-like level, whose relative abundances were significantly different (p-value <0.05 in ANOVA) between the non-secretor genotype AA and the secretor genotypes AG and GG by the HITChip analyses.**
(XLSX)

Table S7 **Original HITChip data.**
(XLS)
