# Supplementary material for: Faecal Microbiota Composition in Adults Is Associated with the FUT2 Gene Determining the Secretor Status
Source: PLoS One. 2014 Apr 14;9(4):e94863. doi: 10.1371/journal.pone.0094863 (PMC3986271; doi:10.1371/journal.pone.0094863)
Supplement: Figure S1 — RDA plots of bifidobacteria, lactobacilli, Clostridium cluster IV and XIVa and Bacteroides fragilis populations in the non-secretors (white) and the secretors (black). The RDA analyses were based on PCR-DGGE profiles of the samples. The centroids of each group are indicated by triangles. P-values show statistical significance in ANOVA test. (PDF) [file pone.0094863.s001.pdf]

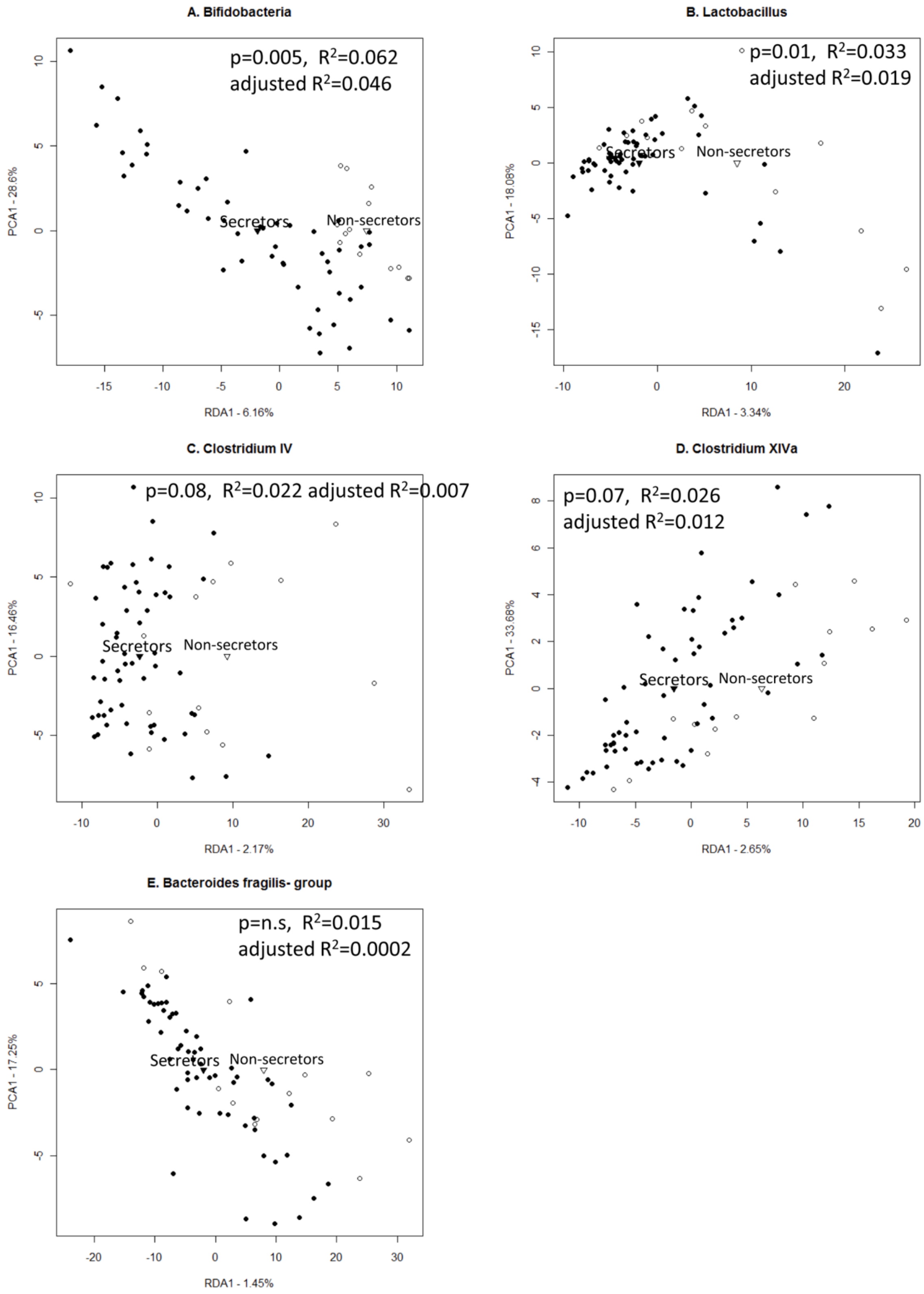

Figure S1. RDA plots of bifidobacteria, lactobacilli, *Clostridium* cluster IV and XIVa and *Bacteroides fragilis* populations in the non-secretors (white) and the secretors (black). The RDA analyses were based on PCR-DGGE profiles of the samples. The centroids of each group are indicated by triangles. P-values show statistical significance in ANOVA test.
