# Supplementary material for: Faecal Microbiota Composition in Adults Is Associated with the FUT2 Gene Determining the Secretor Status
Source: PLoS One. 2014 Apr 14;9(4):e94863. doi: 10.1371/journal.pone.0094863 (PMC3986271; doi:10.1371/journal.pone.0094863)
Supplement: Figure S2 — RDA plots of bifidobacteria, lactobacilli, Clostridium cluster IV and XIVa and Bacteroides fragilis populations in the individuals with FUT2 genotypes AA (white), AG (grey) and GG (black). The RDA analysis based on the PCR-DGGE profiles of the samples. The centroids of each group are indicated by triangles. P-values show statistical significance in ANOVA test. (PDF) [file pone.0094863.s002.pdf]

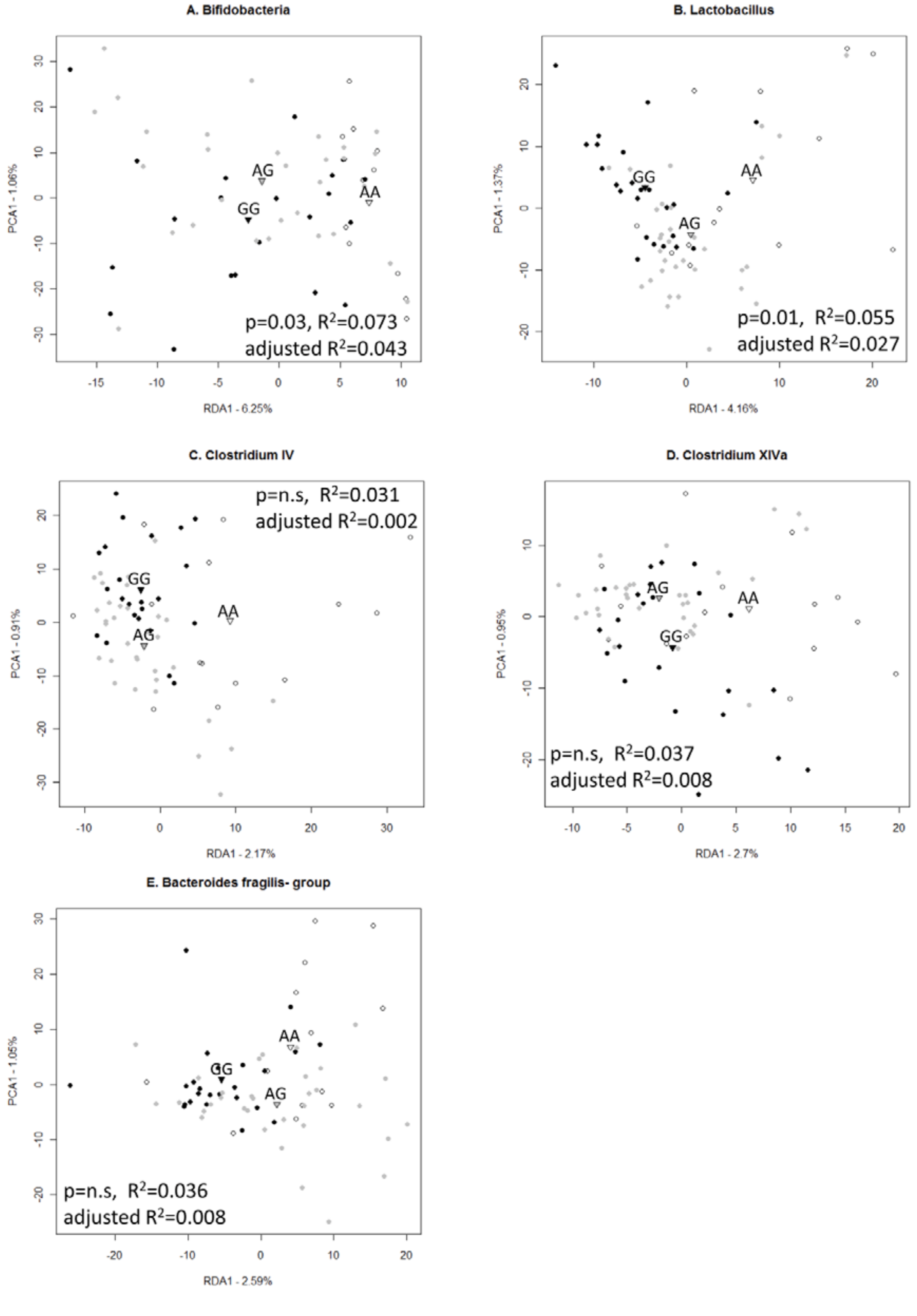

Figure S2. RDA plots of bifidobacteria, lactobacilli, *Clostridium* cluster IV and XIVa and *Bacteroides fragilis* populations in the individuals with FUT2 genotypes AA (white), AG (grey) and GG (black). The RDA analysis based on the PCR-DGGE profiles of the samples. The centroids of each group are indicated by triangles. P-values show statistical significance in ANOVA test.
