# Supplementary material for: Faecal Microbiota Composition in Adults Is Associated with the FUT2 Gene Determining the Secretor Status
Source: PLoS One. 2014 Apr 14;9(4):e94863. doi: 10.1371/journal.pone.0094863 (PMC3986271; doi:10.1371/journal.pone.0094863)
Supplement: Figure S3 — MDS plots of intestinal microbiota compositions in the individuals with FUT2 non-secretor genotype AA (white), secretor genotype AG (grey) and GG (black) based on band intensities of the DGGE analysis (A), on abundances of genera by pyrosequencing (B), on abundances of level 2 taxa by HITChip (C), on abundances of OTUs by pyrosequencing (D) and on abundances of genera in subsampled pyrosequencing data. (PDF) [file pone.0094863.s003.pdf]

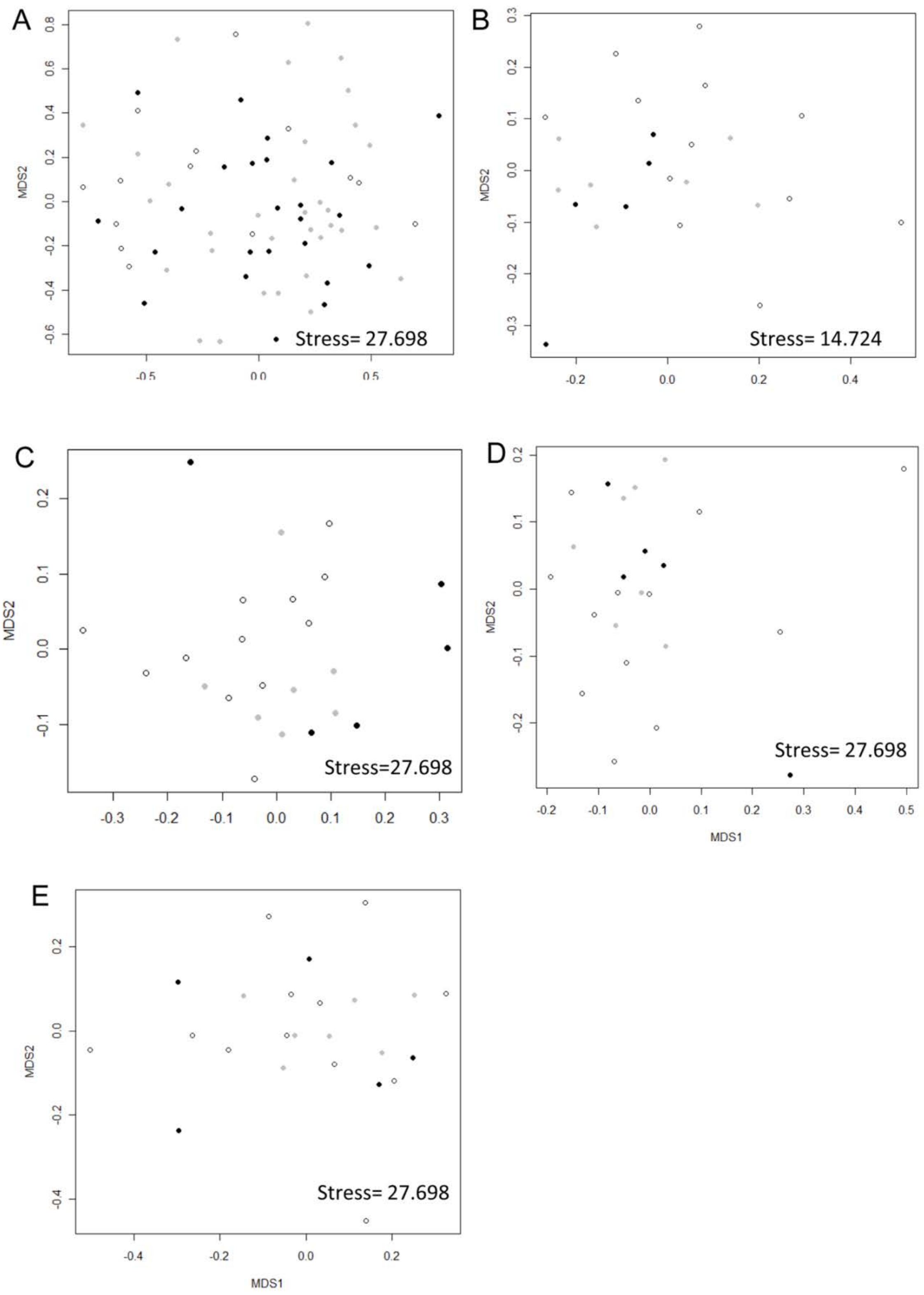

Figure S3. MDS plot of intestinal microbiota compositions in the individuals with FUT2 non-secretor genotype AA (white), secretor genotype AG (grey) and GG (black) based on band intensities of the DGGE analysis (A), on abundances of genera by pyrosequencing (B), on abundances of level 2 taxa by HITChip (C), on abundances of OTUs by pyrosequencing (D) and on abundances of genera in subsampled pyrosequencing data.
