# Supplementary material for: Faecal Microbiota Composition in Adults Is Associated with the FUT2 Gene Determining the Secretor Status
Source: PLoS One. 2014 Apr 14;9(4):e94863. doi: 10.1371/journal.pone.0094863 (PMC3986271; doi:10.1371/journal.pone.0094863)
Supplement: Figure S4 — Rarefaction curves for the non-secretor and the secretor samples based on detected OTUs using 0.97 similarity threshold (A) and on the genera (B). Blue line = non-secretors/FUT2 genotype AA, red line = FUT2 genotype AG, green line = FUT2 genotype GG. (PDF) [file pone.0094863.s004.pdf]

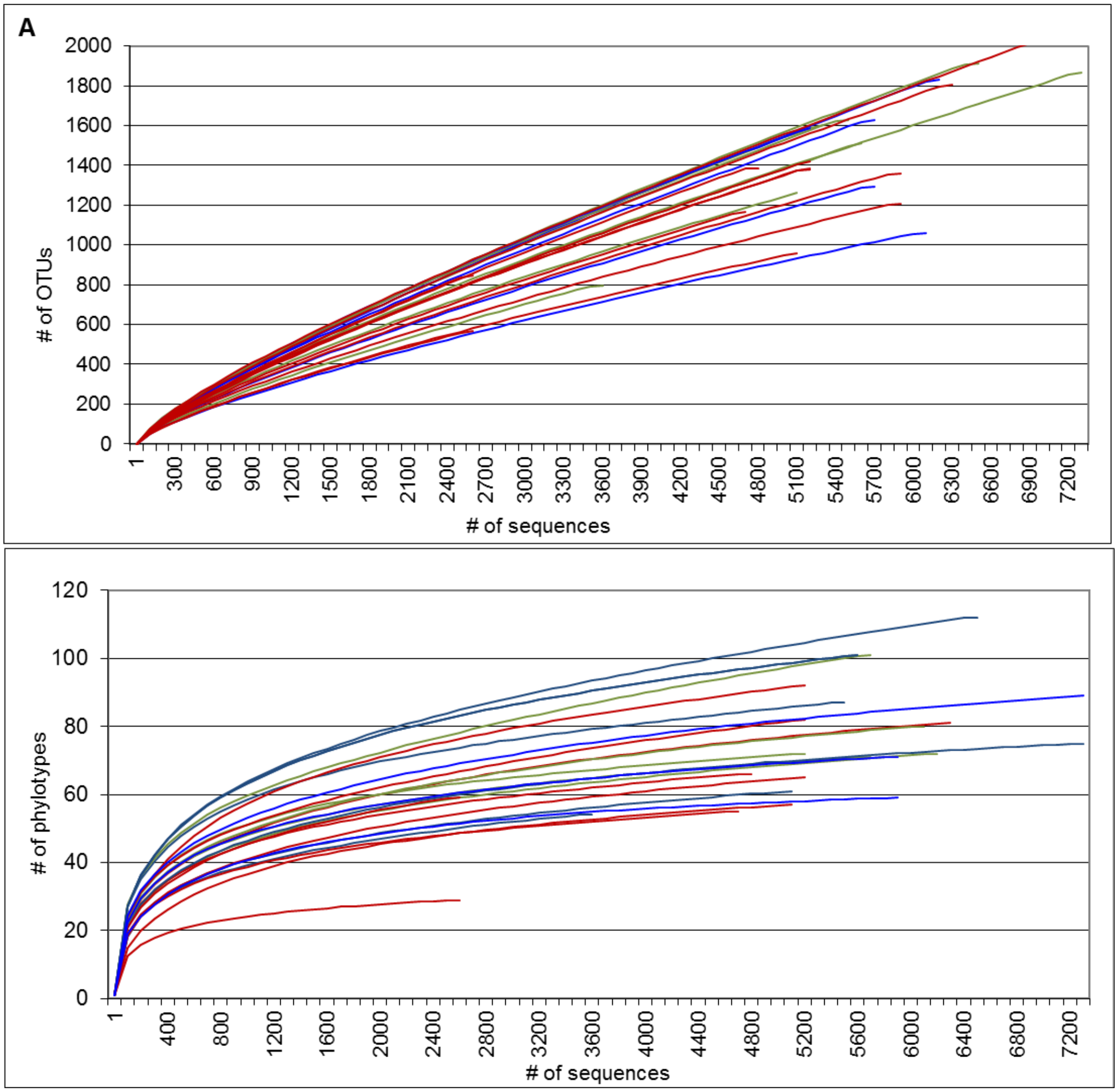

Figure S4. Rarefaction curves for the non-secretor and the secretor samples based on detected OTUs using 0.97 similarity threshold (A) and on the genera (B). Blue lines= non-secretors/FUT2 genotype AA, Red lines=FUT2 genotype AG; green line= FUT2 genotype GG.
