# Supplementary material for: Faecal Microbiota Composition in Adults Is Associated with the FUT2 Gene Determining the Secretor Status
Source: PLoS One. 2014 Apr 14;9(4):e94863. doi: 10.1371/journal.pone.0094863 (PMC3986271; doi:10.1371/journal.pone.0094863)
Supplement: Figure S5 — RDA plots based on the OTUs detected in the non-secretors (white) and the secretors (black) (A) and among the FUT2 genotypes, AA (white), AG (grey) and GG (black) (B). Threshold of 97% similarity was used for clustering of the sequences into OTUs. The centroids of each group are indicated by triangles. P-values show statistical significance in ANOVA test. (PDF) [file pone.0094863.s005.pdf]

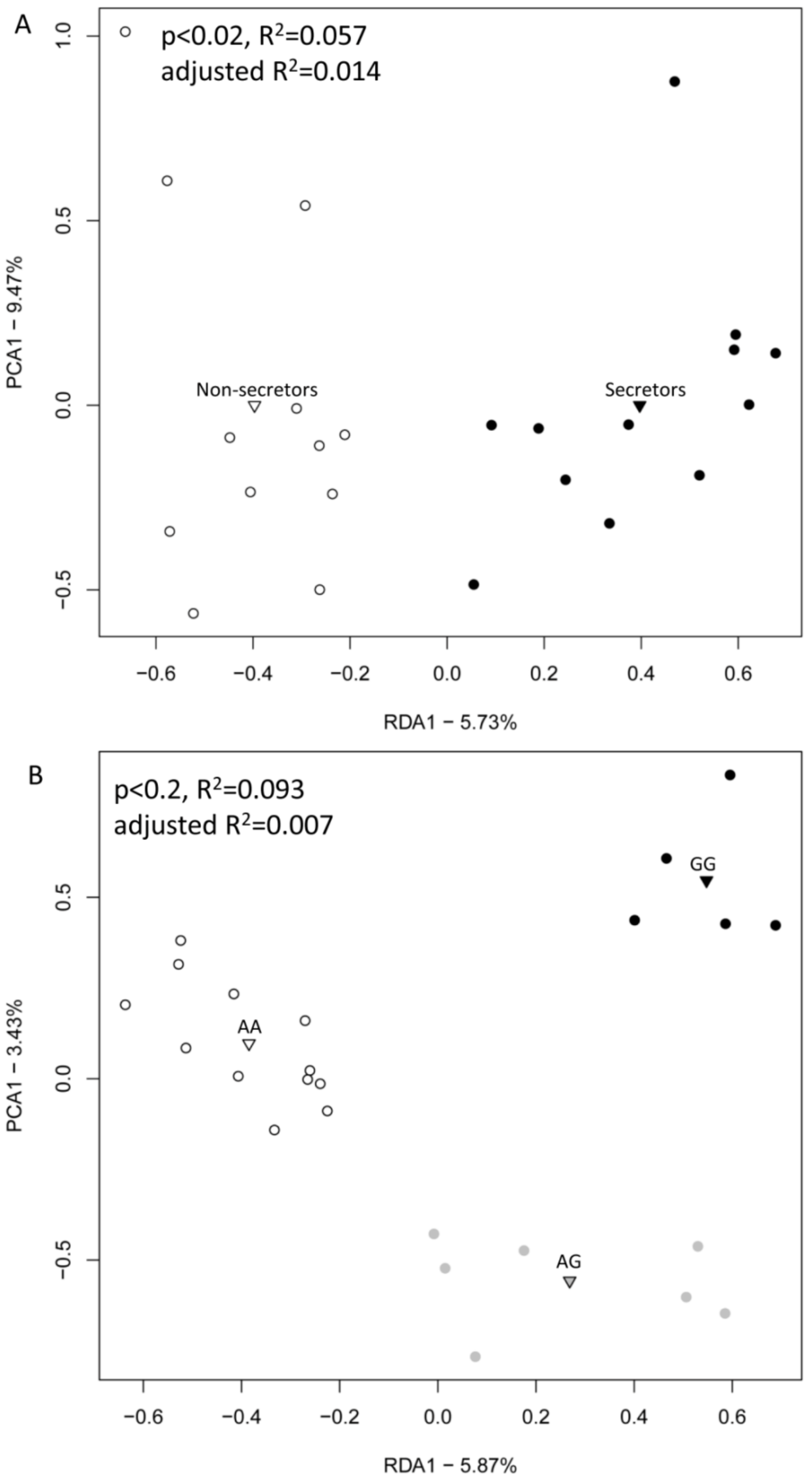

Figure S5. RDA plot based on the OTUs detected in the non-secretors (white) and the secretors (black) (A) and among the *FUT2* genotypes, AA (white), AG (grey) and GG (black) (B). Threshold of 97% similarity was used for clustering of the sequences into OTUs. The centroids of each group are indicated by triangles. P-values show statistical significance in ANOVA test.
