# Supplementary material for: Faecal Microbiota Composition in Adults Is Associated with the FUT2 Gene Determining the Secretor Status
Source: PLoS One. 2014 Apr 14;9(4):e94863. doi: 10.1371/journal.pone.0094863 (PMC3986271; doi:10.1371/journal.pone.0094863)
Supplement: Figure S6 — RDA plots of microbiota compositions at genus level in the non-secretors (white) and the secretors (black) (A) and among the FUT2 genotypes, AA (white), AG (grey) and GG (black) (B). Plots are based on random subsample of the pyrosequencing data set (2500 sequences per sample, total 60 000 sequences). The centroids of each group are indicated by triangles. P-values show statistical significance in ANOVA test. (PDF) [file pone.0094863.s006.pdf]

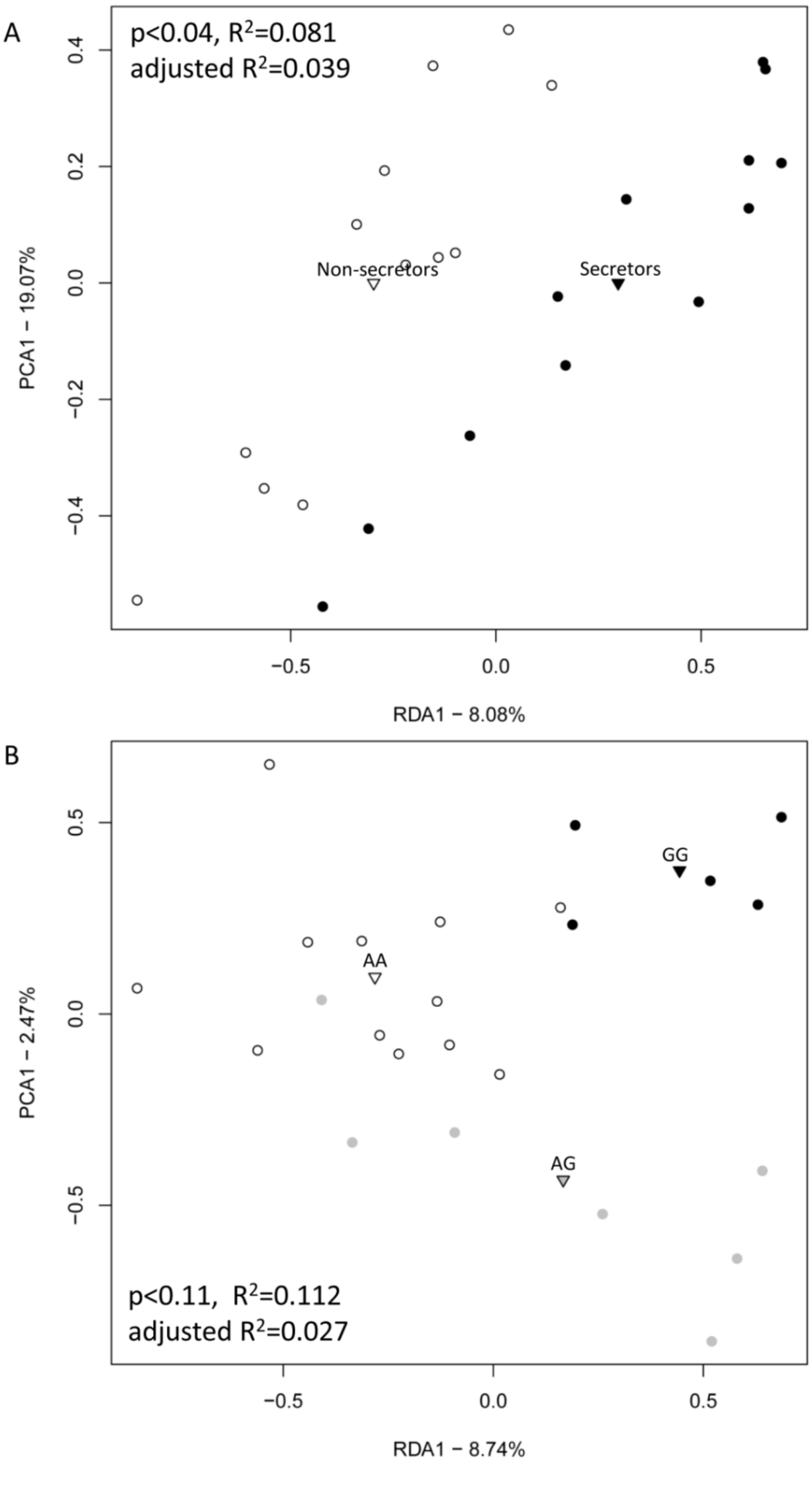

Figure S6. RDA plot of microbiota composition at genus level in the non-secretors (white) and the secretors (black) (A) and among the *FUT2* genotypes, AA (white), AG (grey) and GG (black) (B). Plots are based on random subsample of the pyrosequencing data set (2500 sequences per sample, total 60 000 sequences). The centroids of each group are indicated by triangles. P-values show statistical significance in ANOVA test.
