# Supplementary material for: Faecal Microbiota Composition in Adults Is Associated with the FUT2 Gene Determining the Secretor Status
Source: PLoS One. 2014 Apr 14;9(4):e94863. doi: 10.1371/journal.pone.0094863 (PMC3986271; doi:10.1371/journal.pone.0094863)
Supplement: Figure S7 — Relative abundances of bacterial phyla in the secretors/ FUT2 genotypes AG (n = 7) and GG (n = 5) and in the non-secretors/genotype AA (n = 12). The abundances were based on the 16S rRNA gene pyrosequencing. (PDF) [file pone.0094863.s007.pdf]

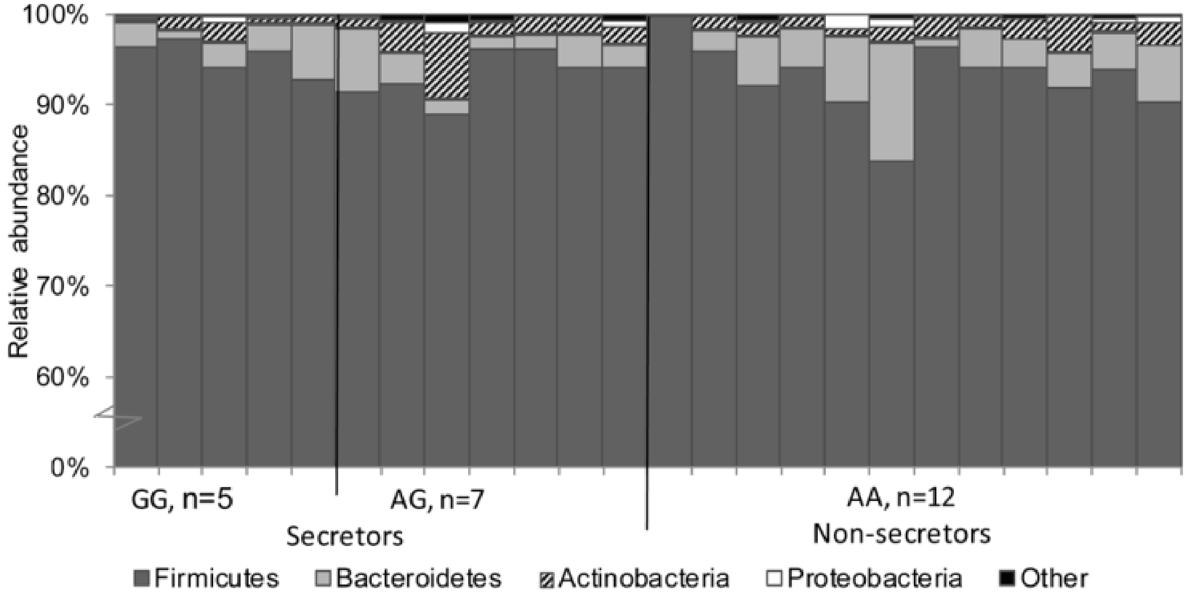

Figure S7. Relative abundances of bacterial phyla in the secretors/*FUT2* genotypes AG (n=7) and GG (n=5) and in the non-secretors/genotype AA (n=12). The abundances were based on the 16S rRNA gene pyrosequencing.

.
