# Supplementary material for: Faecal Microbiota Composition in Adults Is Associated with the FUT2 Gene Determining the Secretor Status
Source: PLoS One. 2014 Apr 14;9(4):e94863. doi: 10.1371/journal.pone.0094863 (PMC3986271; doi:10.1371/journal.pone.0094863)
Supplement: Figure S8 — RDA plots of intestinal microbiota compositions in the non-secretors (white) and the secretors (black) (A) and among individuals with the FUT2 genotypes AA (white), AG (grey) and GG (black) (B). Plots were based on Hellinger transformed level 3/species-like taxa obtained by HITChip analysis. The triangles indicate centroids of the study groups. P-values show statistical significance in ANOVA test. (PDF) [file pone.0094863.s008.pdf]

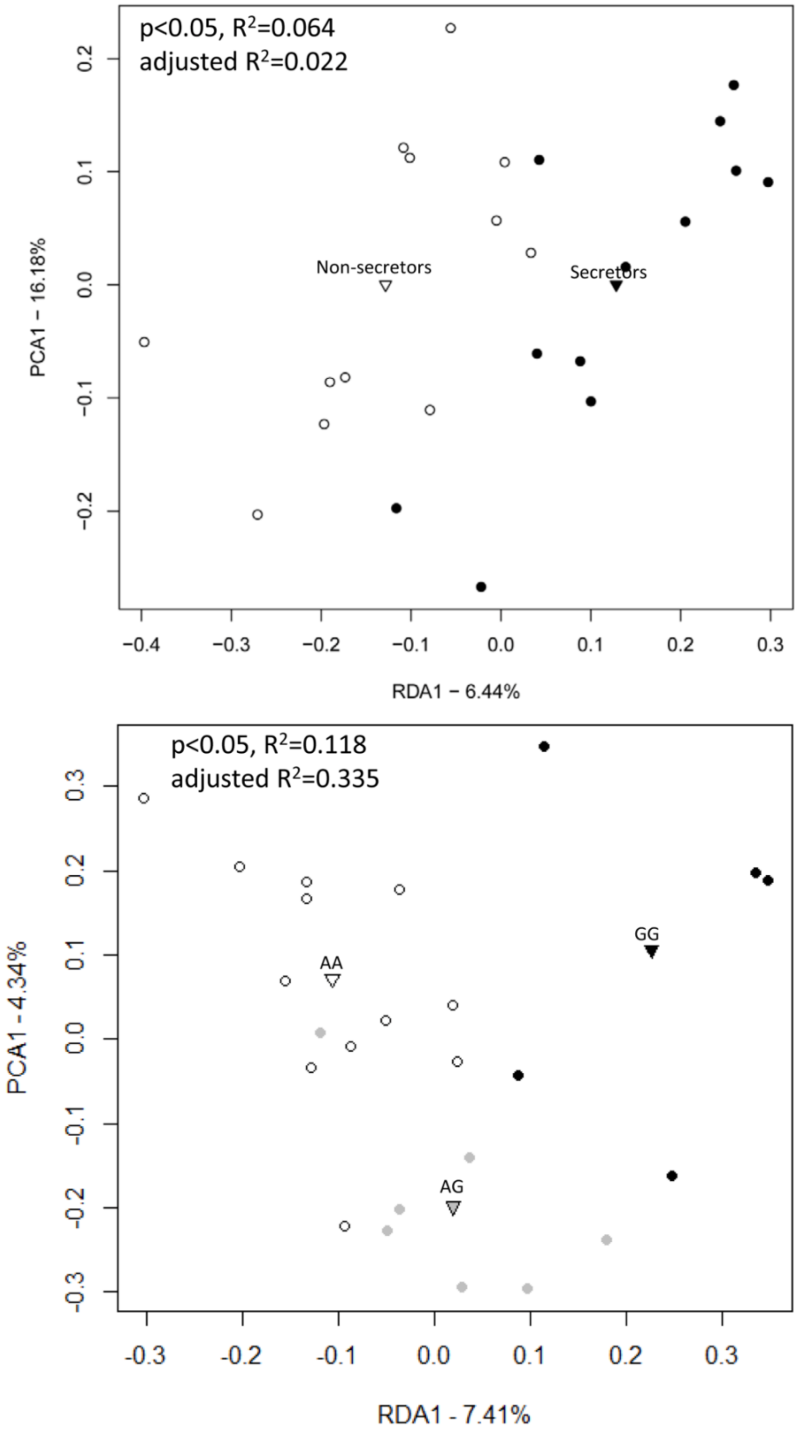

Figure S8. RDA plot of intestinal microbiota compositions in the non-secretors (white) and the secretors (black) (A) and among individuals with the *FUT2* genotypes AA (white), AG (grey) and GG (black) (B). Plots were based on Hellinger transformed level 3/species-like taxa obtained by HITChip analysis. The triangles indicate centroids of the study groups. P-values show statistical significance in ANOVA test.
