# Supplementary material for: Faecal Microbiota Composition in Adults Is Associated with the FUT2 Gene Determining the Secretor Status
Source: PLoS One. 2014 Apr 14;9(4):e94863. doi: 10.1371/journal.pone.0094863 (PMC3986271; doi:10.1371/journal.pone.0094863)
Supplement: Figure S9 — Bacterial diversity in the individuals with FUT2 non-secretor genotype AA (n = 12), and with secretor genotypes AG (n = 7) and GG (n = 5). The results were based on the HITChip analysis. (PDF) [file pone.0094863.s009.pdf]

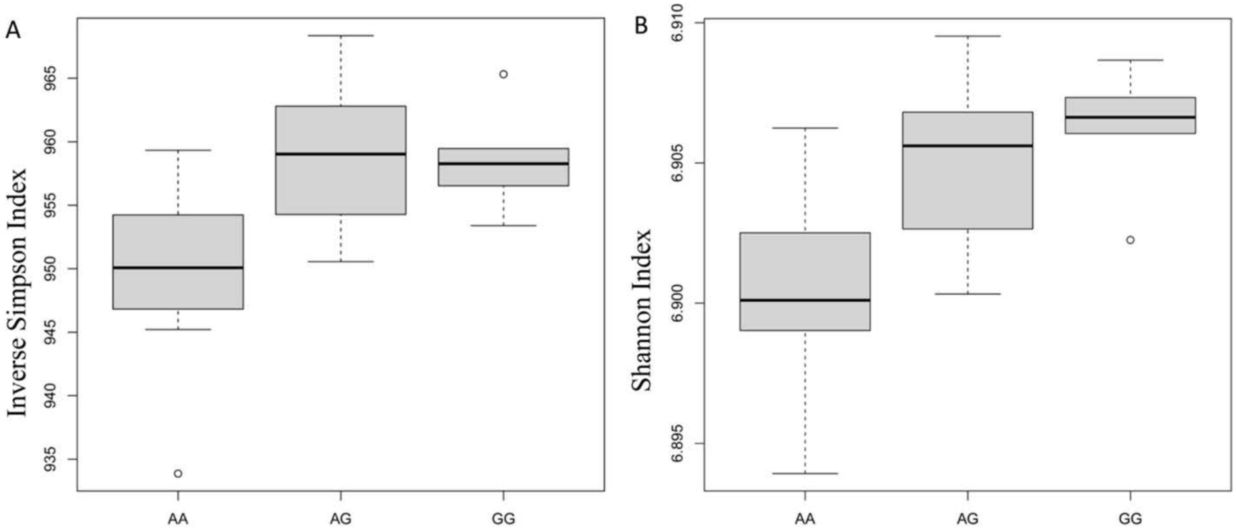

Figure S9. Inverse Simpson diversity index (A) and Shannon diversity index (B) in the individuals with *FUT2* non-secretor genotype AA (n=12), and with secretor genotypes AG (n=7) and GG (n=5). The results were based on the HITChip analysis.
