# Supplementary material for: Faecal Microbiota Composition in Adults Is Associated with the FUT2 Gene Determining the Secretor Status
Source: PLoS One. 2014 Apr 14;9(4):e94863. doi: 10.1371/journal.pone.0094863 (PMC3986271; doi:10.1371/journal.pone.0094863)
Supplement: Figure S10 — ANOVA p-value histogram for level 3/species –like level differences between the non-secretor and the secretors (A) and different FUT2 genotypes (B) in HITChip analysis. (PDF) [file pone.0094863.s010.pdf]

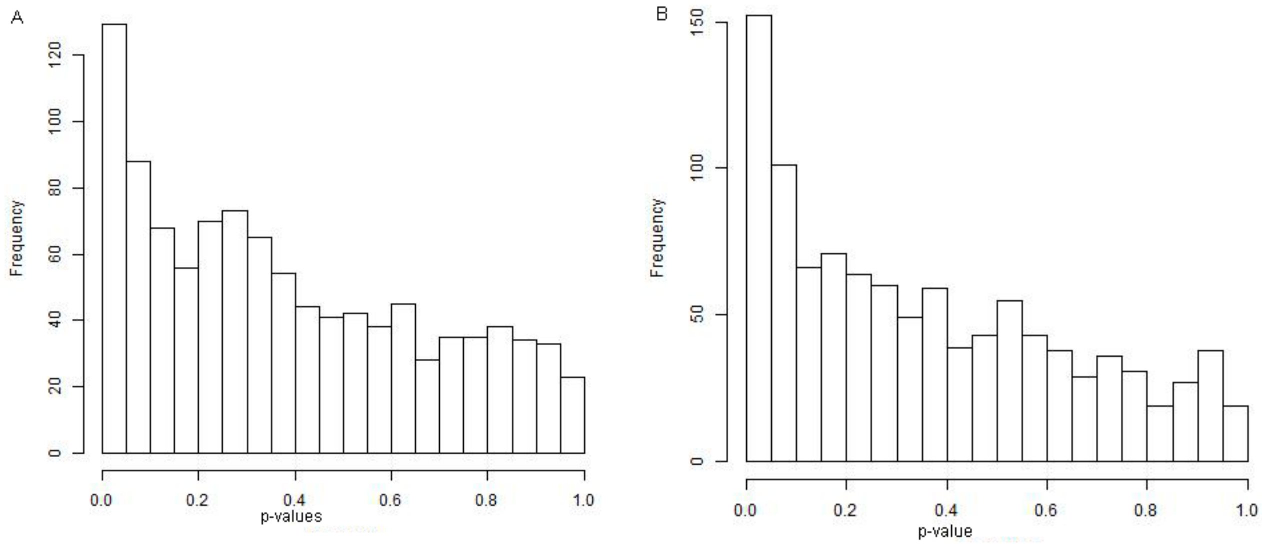

Figure S10. ANOVA p-value histogram for le 3/el species –like level differences between the non-secretor and the secretors (A) and different genotypes (B) in HITChip analysis.
