# Supplementary material for: Faecal Microbiota Composition in Adults Is Associated with the FUT2 Gene Determining the Secretor Status
Source: PLoS One. 2014 Apr 14;9(4):e94863. doi: 10.1371/journal.pone.0094863 (PMC3986271; doi:10.1371/journal.pone.0094863)
Supplement: Figure S11 — Co-occurrences based on the relative abundances of the level 2/genus-like level bacterial taxa for the secretors (A) and the non-secretors (B). Co-occurrences with ρ>0.7, relative abundance of the bacterial group >0.1%, and present >50% of the non-secretor or secretor samples are shown. Light green indicate positive and yellow negative co-occurrences. Statistically significant positive and negative co-occurrences (q-value <0.05) are shown in blue and red, respectively. (PDF) [file pone.0094863.s011.pdf]

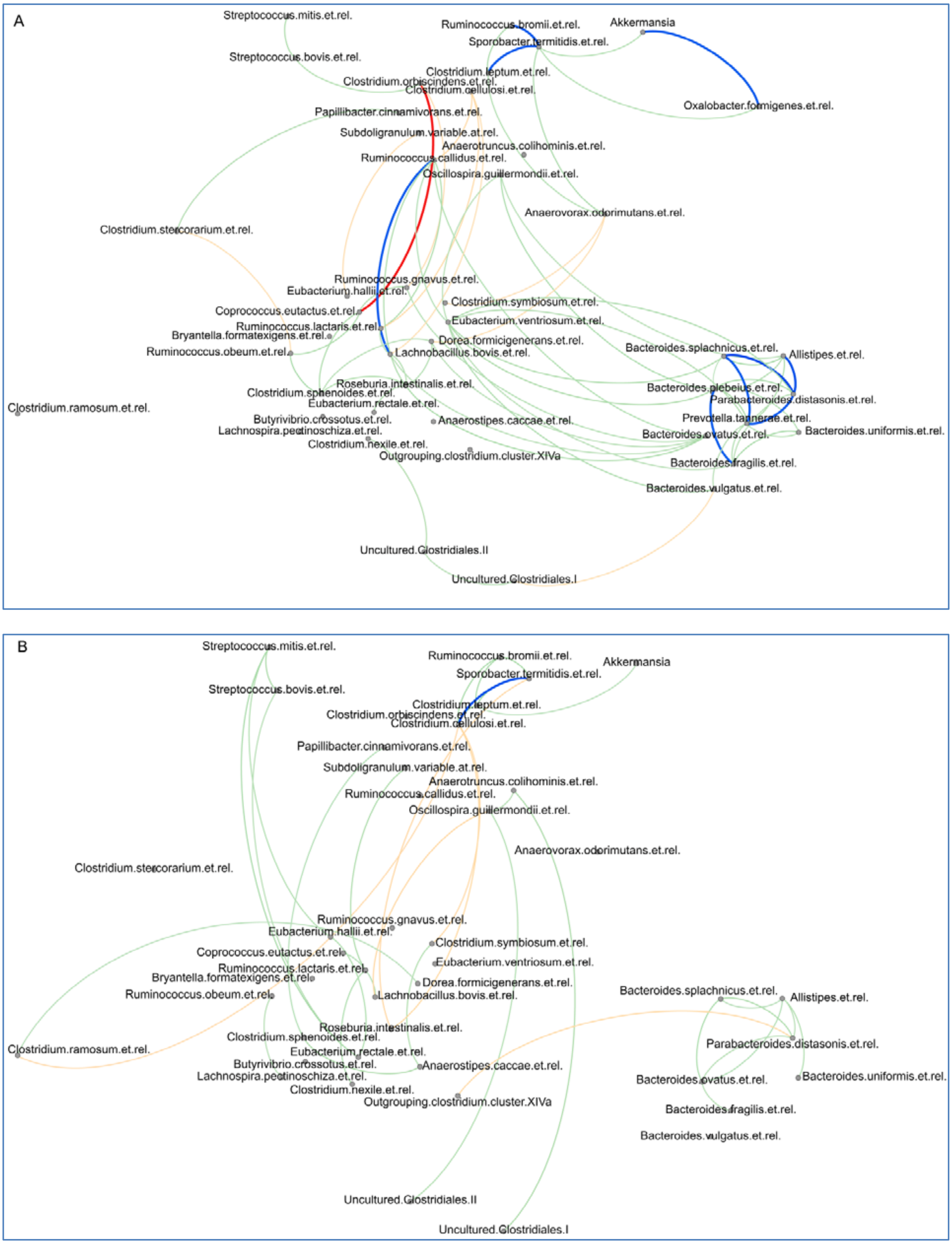

Figure S11. Co-occurrences based on the relative abundances of the level 2/genus-like level bacterial taxa for the secretors (A) and the non-secretors (B). Co-occurrences with $\rho > 0.7$, relative abundance of the bacterial group >0.1%, and present >50% of the non-secretors or secretors samples are shown. Light green indicate positive and yellow negative co-occurrences. Statistically significant positive and negative co-occurrences (q-value <0.05) are shown in blue and red, respectively.
