# Supplementary material for: Faecal Microbiota Composition in Adults Is Associated with the FUT2 Gene Determining the Secretor Status
Source: PLoS One. 2014 Apr 14;9(4):e94863. doi: 10.1371/journal.pone.0094863 (PMC3986271; doi:10.1371/journal.pone.0094863)
Supplement: Table S1 — The number of the 16S rRNA gene sequences obtained from the non-secretor samples, the secretor samples and the samples with different FUT2 genotypes by pyrosequencing. (PDF) [file pone.0094863.s012.pdf]

Table S1. The number of the 16S rRNA gene sequences obtained from the non-secretor samples, the secretor samples and the samples with different FUT2 genotypes by pyrosequencing.

	**Non-secretor (AA)**	**Secretor (AG/GG)**	**Secretor, AG**	**Secretor, GG**	**Total**
**Samples**	12	12	7	5	24
**total # of Reads**	118 507	127 299	72 843	54 456	245 806
**# of reads passing QC**	60 184	67 168	38 532	28 636	127 352
**Average # (range) of reads per sample**	5 015 (2529 - 7276)	5 597 (3509 - 7257)	5505 (3509-7257)	5727 (5187-6137)	5306 (2529-7276)
**Good's coverage**	0.796	0.787	0.733	0.794	0.792
