# Supplementary material for: Faecal Microbiota Composition in Adults Is Associated with the FUT2 Gene Determining the Secretor Status
Source: PLoS One. 2014 Apr 14;9(4):e94863. doi: 10.1371/journal.pone.0094863 (PMC3986271; doi:10.1371/journal.pone.0094863)
Supplement: Table S2 — Average relative abundances (%) of enterotype indicator species in the non-secretors, the secretors and the individuals with FUT2 genotypes AA, AG or GG belonging to the enterotype 1 (ET1) or 3 (ET3). (PDF) [file pone.0094863.s013.pdf]

Table S2. Average relative abundances (%) of enterotype indicator species in the non-secretors, the secretors and the individuals with FUT2 genotypes AA, AG or GG belonging to the enterotype 1 (ET1) or 3 (ET3).

**Taxa**	**Non-secretors (ET1)**	**Genotype AG (ET1)**	**Non-secretors (ET3)**	**Secretors (ET3)**	**Genotype AG (ET3)**	**Genotype GG (ET3)**
Bacteroides fragilis et rel.	1.7	0.6	0.6	1.2	1.4	0.9
Bacteroides ovatus et rel.	1.7	1.1	0.6	1.2	1.3	1.1
Bacteroides vulgatus et rel.	1.3	1.6	0.7	1.7	1.9	1.4
Clostridium symbiosum et rel.	4.0	4.4	3.1	2.0	2.4	1.6
Roseburia intestinalis et rel.	3.8	4.0	2.0	1.4	1.8	0.9
Ruminococcus bromii et rel.	1.1	0.1	2.7	3.3	2.7	3.8
Ruminococcus callidus et rel.	0.9	2.2	1.7	1.4	1.9	0.9
Sporobacter termitidis et rel.	0.3	0.6	1.1	1.6	1.3	1.8
Tannerella et rel.	0.4	0.3	1.2	1.3	1.9	0.7
Uncultured Clostridiales I	0.1	0.01	2.5	5.5	2.8	8.2
