# Supplementary material for: Faecal Microbiota Composition in Adults Is Associated with the FUT2 Gene Determining the Secretor Status
Source: PLoS One. 2014 Apr 14;9(4):e94863. doi: 10.1371/journal.pone.0094863 (PMC3986271; doi:10.1371/journal.pone.0094863)
Supplement: Table S3 — The bacterial level 1 and 2 taxa, whose relative abundances were significantly different (p-value <0.05 in ANOVA) between the non-secretor (NSS) and the secretor (SS) individuals by the HITChip analyses. (PDF) [file pone.0094863.s014.pdf]

Table S3. The bacterial level 1 and 2 taxa, whose relative abundances were significantly different (p-value < 0.05 in ANOVA) between the non-secretor (NSS) and the secretor (SS) individuals by the HITChip analyses.

**Level**	**Taxa**		**Direction**	**p-value**
**Level 1**				
	Clostridium cluster I	Firmicutes	SS>NSS	0.04
	Clostridium cluster XIVa	Firmicutes	NSS>SS	0.0009
**Level 2**				
	Eggerthella lenta et rel.	Actinobacteria	NSS>SS	0.006
	Clostridia	Clostridium cluster I	SS>NSS	0.04
	Anaerotruncus colihominis et rel.	Clostridium cluster IV	SS>NSS	0.03
	Dorea formicigenerans et rel.	Clostridium cluster XIVa	NSS>SS	0.001
	Lachnospira pectinoschiza et rel.	Clostridium cluster XIVa	NSS>SS	0.001
	Outgrouping clostridium cluster XIVa	Clostridium cluster XIVa	NSS>SS	0.03
	Ruminococcus gnavus et rel.	Clostridium cluster XIVa	NSS>SS	0.003
	Blautia	Clostridium cluster XIVa	NSS>SS	0.03
	Clostridium ramosum et rel.	Clostridium cluster XVIII	NSS>SS	0.02
	Christensenella et rel.	Uncultured Clostridiales	SS>NSS	0.009
