# Supplementary material for: Faecal Microbiota Composition in Adults Is Associated with the FUT2 Gene Determining the Secretor Status
Source: PLoS One. 2014 Apr 14;9(4):e94863. doi: 10.1371/journal.pone.0094863 (PMC3986271; doi:10.1371/journal.pone.0094863)
Supplement: Table S4 — The bacterial level 1 and 2 taxa, whose were relative abundances were significantly different between the samples with different FUT2 genotypes based on HIT Chip analyses. (PDF) [file pone.0094863.s015.pdf]

Table S4. The bacterial level 1 and 2 taxa, whose were relative abundances were significantly different between the samples with different FUT2 genotypes based on HIT Chip analyses.

**Taxa**		**Direction**	**p-value, AA vs AG**	**p-value, AA vs GG**
**Level 1**				
Clostridium cluster I	Firmicutes	GG>AA	ns	0.004
Clostridium cluster IV	Firmicutes	GG>AA	ns	0.006
Clostridium cluster XI	Firmicutes	GG>AA	ns	0.01
Clostridium cluster XIII	Firmicutes	GG>AA	ns	0.04
Clostridium cluster XIVa	Firmicutes	GG>AG>AA	0.006	0.005
Lentisphaerae	Chlamydiae/Verrucomicrobia group	GG>AA	ns	0.007
**Level 2**				
Anaerovorax odorimutans et rel.	Clostridium cluster XI	GG>AA	ns	0.02
Clostridium cellulosi et rel.	Clostridium cluster IV	GG>AA	ns	0.0001
Clostridium difficile et rel.	Clostridium cluster XI	GG>AA	ns	0.008
Dorea formicigenerans et rel. *	Clostridium cluster XIVa	AA>AG>GG	0.01	0.002
Eggerthella lenta et rel.*	Actinobacteria	AA>GG>AG	0.007	0.09
Eubacterium hallii et rel.	Clostridium cluster XIVa	AA>AG>GG	0.02	ns
Lachnospira pectinoschiza et rel.*	Clostridium cluster XIVa	AA>AG>GG	0.008	0.005
Peptostreptococcus micros et rel.	Clostridium cluster XIII	GG>AA	ns	0.04
Ruminococcus gnavus et rel.*	Clostridium cluster XIVa	AA>AG>GG	0.01	0.01
Uncultured Clostridiales II*	Uncultured Clostridiales	GG>AG>AA	0.07	0.01
Clostridia*	Clostridium cluster I	GG>AA	ns	0.004
Corynebacterium	Actinobacteria	GG>AA	ns	0.005
Propionibacterium	Actinobacteria	GG>AA	ns	0.002
